# Motor-evoked potentials in amyotrophic lateral sclerosis: potential implications in detecting subclinical UMN involvement in lower motor neuron phenotype

**DOI:** 10.1007/s00415-020-10073-5

**Published:** 2020-07-16

**Authors:** Stefano Zoccolella, Antonella Mastronardi, Antonio Scarafino, Giovanni Iliceto, Eustachio D’Errico, Angela Fraddosio, Irene Tempesta, Antonella Morea, Gaspare Scaglione, Alessandro Introna, Isabella Laura Simone

**Affiliations:** 1 Neurology Unit, ASL Bari, San Paolo Hospital, Bari, Italy; 2 Department of Basic Medical Sciences, Sense Organs and Neurosciences, Neurology Unit, University of Bari “Aldo Moro”, Piazza Giulio Cesare 11, 70124 Bari, Italy; 3 Neurology Unit, “Miulli” Hospital, Acquaviva Delle Fonti, Bari, Italy

**Keywords:** Motor-evoked potentials, Amyotrophic lateral sclerosis, Lower motor neuron

## Abstract

**Background:**

In amyotrophic lateral sclerosis (ALS), the involvement of lower motor neuron is well defined by electromyography, whereas a reliable marker of upper motor neuron (UMN) damage still lacks. Aim of the study was to estimate the role of transcranial magnetic stimulation (TMS)-induced motor-evoked potentials (MEPs) as marker of subclinical UMN involvement.

**Methods:**

Clinical evidence of UMN damage was prospectively compared to MEPs in 176 ALS patients diagnosed between 2011 and 2014, and classified according to existing diagnostic criteria. Finally, we evaluated the appearance of clinical UMN signs and the level of diagnostic certainty in ALS after 1 year of follow-up.

**Results:**

At presentation, abnormal MEPs were found in 80% of patients with clinical evidence of UMN damage and in 72% of patients without clinical involvement of UMN. Among these latter, 61% showed appearance of UMN clinical signs after 1 year. Approximately 70% of patients with clinical lower motor neuron (LMN) phenotype showed MEP abnormalities, while they were considered not classifiable ALS according to Airlie house or Awaji criteria. Furthermore, abnormal MEPs in absence of clinical UMN signs at baseline were found in 80% of spinal ALS that after 1-year developed UMN signs at limbs, compared to 50% of bulbar ALS.

**Conclusions:**

TMS is a reliable marker of subclinical UMN damage particularly among LMN phenotype and ensure an early ALS diagnosis in ~ 70% of such cases.

## Introduction

Amyotrophic lateral sclerosis (ALS) is a fatal neurodegenerative disease characterized by upper (UMN) and lower motor neurons (LMN) damage. Given the clinical heterogeneity and the prevalence of atypical phenotypes, the diagnostic delay from symptom onset in ALS is commonly12 months [1].

The diagnosis of ALS is primarily clinical, and requires the careful exclusion of ALS-mimic syndromes [2]. Electrophysiological studies, especially needle electromyography, can recognize LMN involvement based on the evidence of either active or chronic denervation and fasciculation potentials, even in the early stage of the disease [3]. Therefore, to improve the diagnostic power of clinically based El Escorial criteria (EEC) [2], electromyographic (EMG) features have been incorporated in Airlie house criteria (AHC) and more recently in Awaji criteria (AC), that consider EMG signs equal to the clinical evidence of LMN impairment [4–6].

On the other hand, the lack of a reliable marker of UMN damage beyond the clinical evidence still remains a critical issue. Transcranial magnetic stimulation (TMS)-induced motor evoked potentials (MEPs) represents a non-invasive method for assessing central motor pathway damage during the course of ALS [7]. Although TMS has been considered as a sensitive test for UMN involvement in ALS, there is still no consensus on its sensitivity, especially for patients without or with doubtful UMN signs [8].

The aim of this study is to evaluate the feasibility in using TMS alterations indicative of UMN impairment in clinical practice in a cohort of ALS patients, to improve the diagnostic certainty level of ALS.

## Materials and methods

### Patients’ selection

We prospectively evaluated 176 incident patients with a diagnosis of sporadic ALS referring at our ALS tertiary center from the Apulia ALS registry [9] in the period 1 January 2011–30 December 2014.

ALS diagnosis was confirmed in all patients after exclusion of mimicking diseases. All patients underwent clinical evaluation by a neurologist of the ALS team using a standardized search for UMN and LMN signs in the four clinical region defined by ALS clinical criteria of El Escorial [2]. In particular, increased or clonic tendon reflexes, spasticity, pseudobulbar features, clonic jaw, gag reflex, exaggerated snout reflex, forced yawing, loss of superficial abdominal reflexes, Hoffmann reflex and extensor plantar response were considered as signs of UMN involvement [2]. The presence of one or more of the aforementioned signs in one body region was considered as clinical indicator of UMN involvement [2].

All patients were classified according to available diagnostic criteria (EEC, AHC, and AC) at baseline and according to EEC criteria at 1-year follow-up visit. For the purpose of the study at baseline, we included patients classifiable as definite, probable, possible, and suspected ALS according to EEC. Paraclinical evaluation included EMG, nerve conduction studies (NCS), TMS, MRI of the brain and spinal cord, hematological and cerebrospinal fluid analyses. All patients underwent MRI of the brain and spinal cord. CSF analysis was performed in carefully selected patients to fully exclude other diagnosis (~ 40% of cases).

At time of TMS, all patients assumed riluzole from at least 1 month, and/or other ion channel targeted drugs (as pregabalin, gabapentin, or some antiarrhythmic or antihypertensive drugs), without any suspension of the drugs during the study period. No patient did not undergo TMS for intolerance, biomedical devices, or history of seizures. For the design of the study, a second clinical evaluation was considered after 1 year from the presentation. No patient was lost at 1-year follow-up and and no alternative diagnosis was given at that time. The study was approved by the Interregional Independent Ethical Committee of “Azienda Ospedaliero-Universitaria of Bari”-Italy. Written informed consent was obtained by subjects participating in the study.

### Electrophysiological investigation

NCS and needle EMG were performed at presentation according to established techniques by experienced neurophysiologists [10]. Special attention was given to the detection of fasciculation potentials. In all patients, four body regions were sampled with EMG (bulbar, cervical, thoracic, and lumbosacral) and in EMG reports, the number of affected regions according to AHC and AC were recorded [4, 5]. Motor conduction studies and F waves were evaluated on ulnar and median nerves for upper limbs and tibial and peroneous nerves on lower limbs. Sensory conduction studies were performed bilaterally on median nerve at upper limbs and sural nerve at lower limbs.

A Magstim super rapid (2 T peak magnetic field) magnetic stimulator was used for MEPs. Stimulation was performed with a focal coil, butterfly shaped (inner diameter 35 mm and outer diameter 190 mm), at the right and left hemispheres above motor areas of hand–foot contralateral to each limb studied, while cervical nerve roots and lumbar plexus ipsilateral to the limb studied. Target muscles were the first dorsal interosseous muscle at upper limbs and the anterior tibial muscle at lower limbs. MEPs were recorded with monopolar 12 × 0.25-mm diameter Ag/AgCl surface electrodes placed on both first interosseous (belly–tendon montage) and tibialis anterior muscles (belly–bone montage). Signals were amplified, stored, and analyzed with a E.B. Neuro-B.E. Light device. Filter settings were 10 Hz–2 kHz. For the recording at upper limbs, cortical stimulation was performed placing the magnetic coil over the brain central areas; the magnetic coil was placed over the vertex for the lower limbs, shifting it more frontally until a maximal response was elicited. Stimulation was performed first at rest, instructing the patient to not activate the target muscle, and then activating the target muscle by minimal voluntary contraction. Cervical stimulation was performed with the magnetic coil placed laterally to the spinal processus C6 for upper limb and L5 for lower limb, with the rim of the coil next to the spinal processus. For each stimulation, the position of the magnetic coil and the intensity of the stimulus were adjusted (when necessary up to the maximum output of the stimulator) to maximize the amplitude of the evoked response, then at least five responses according to MEPs reliability were recorded. Central motor conduction time (CMCT) to each target muscle was calculated by subtracting the latencies upon cervical or lumbar stimulation from the latencies upon cortical stimulation. Absolute MEPs were recorded bilaterally according to the guidelines of the International Federation of Clinical Neurophysiology [11]. MEPs were interpreted by experienced neurophysiologists, blinded for clinical data of the patients (G.I. and S.Z.) with an inter-rater agreement of 90%. In this study, bulbar region was not examined by TMS.

**Fig. 1:**
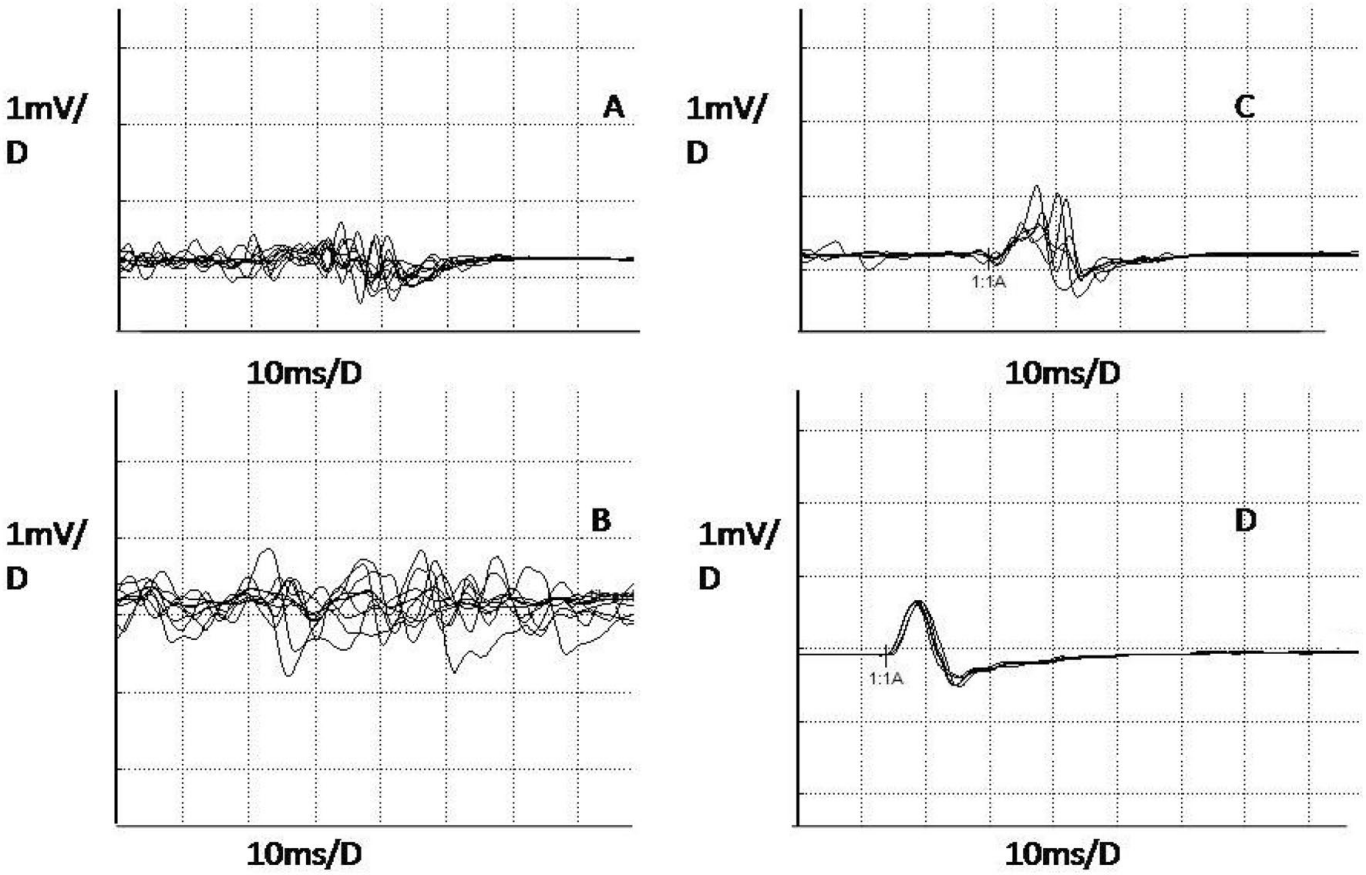
On the left, poorly reliable cortical MEPs from the right first interosseous (**a**) and left tibial anterior (**b**). On the right, cortical MEP (distal latency 29.6 ms, **c**) and peripheral MEP (15 ms, **d**) from left first interosseous, with delayed central motor conduction time (14.6 ms)

Motor latency, amplitude, and central motor conduction time (CMCT) were measured. Cortical silent period was not included in this study as all patients were treated at time of TMS with ion channel targeted drugs (above reported) that may interfere with this parameter. The following electrophysiological features of the UMN function were considered to be abnormal (Fig. 1) (1) absent or unreliable MEPs (Fig. 1a, b), when no motor potential was clearly recognizable at the motor cortex after superimposition of at least ten traces obtained using the maximal output of the stimulator, with normal MEPs following stimulation of motor roots; (2) increased CMCT. In particular, the latency and CMCT were considered abnormal if longer than 18 ms at legs and 8 ms at arms (Fig. 1c, d), based on the mean + 2.5 SD of healthy control group (*n*.70 subjects), matched for sex, age and body height (according to Claus) [12]. Absolute MEPs alterations were defined asymmetric either when unreliable MEP and delayed CMCT were unilateral or when CMCT difference between left and right was ≥ 1.5 ms at upper limbs and ≥ 2 ms at lower limbs.

### Statistical analysis

Demographic and clinical variables were expressed as mean (± SD) or frequencies for continuous and categorical variables. Comparisons between categorical variables were made by Chi square test. Sensitivity, specificity, and predictive values were analyzed. Statistical analysis was performed with IBM SPSS statistics data editor version 22.

## Results

Demographic and clinical features of ALS patients are shown on Table 1. Male/female ratio was 1:3. Spinal onset was more frequent than bulbar onset. Median onset diagnosis interval was 13.2 months. At baseline, patients were classified according to the different three existing ALS diagnostic sets of criteria (EEC, AHC, and AC) (Table 2). Using the EEC criteria, 19% of patients were classified as definite, 34% as probable, 24% as possible, and 23% as suspected ALS, actually LMN phenotype. According to AHC criteria, four patients shifted from possible ALS to “probable-laboratory supported” ALS. Finally, applying the AC criteria, the number of definite and probable ALS diagnosis resulted to be similar to AHC. The forty suspected ALS were not classifiable using both AHC and AC criteria.

**Table 1:** Demographic and clinical data of patients at presentation

	ALS patients *n* 176
Age at presentation, median (range), years	60.8 (17.7–86.0)
Male/female	101/75
Bulbar/spinal onset	43/133
Disease duration from symptom onset to diagnosis, median (range), months	13.21 (0.9–30.78)
Disease duration from symptom onset to presentation, median (range), months	12.81 (2.0–26.40)
Follow-up clinical evaluation, median (range), months	13.2 (8.9–15.3)

**Table 2:** Distribution of patients according to different diagnostic criteria at presentation and according to El Escorial criteria after 1 year of follow-up

Diagnostic category	EEC at presentation *n* of patients	AHC at presentation *n* of patients	AC at presentation *n* of patients	EEC at 1-year follow-up *n* of patients
Definite	33	33	34	81
Probable	60	60	64	48
Probable lab supported	/	4	/	/
Possible	43	39	38	26
Suspected/unclassifiable	40	40 Unclassifiable as ALS	40 Unclassifiable as ALS	21

*EEC* El Escorial criteria, *AHC* Airlie house criteria, *AC* Awaji criteria

We found that 78% (138 out 176) of patients had abnormal MEPs in at least one limb: 57% of patients (*n* = 100) had unreliable MEPs and 36% (*n* = 63) had delayed CMCT. Considering just patients with abnormal MEPs (*n* = 138), delayed CMCT was prevalent at upper limbs (38%, *n* = 52) than at lowers (19%, *n* = 26), whereas unreliable MEP was more frequent in lower limbs (69%, *n* = 95) than in upper (39% *n* = 48). Delayed CMCT and unreliable MEPs were asymmetrical in ~ 30% of cases (*n* = 41 in upper and 45 in lower limbs, respectively); asymmetrical delayed CMCT was more common in upper limbs [23% (*n* = 32)] than in lowers [11% (*n* = 15)], whereas no difference was found in asymmetrical unreliable MEPs distribution among limbs.

When we compared the clinical evidence of UMN impairment at limbs with TMS data, more than 80% (*n* = 102/126) of patients with clinical UMN signs at limbs at presentation had concomitant abnormal MEPs in the same region; on the other hand, more than 70% (*n* = 36/50) of patients without clinical UMN evidence at limbs had abnormal MEPs (*Χ*^2^ = 1.7, *p* = ns, Table 3). Approximately 60% of patients without any clinical UMN signs at limbs and with MEPs abnormalities at baseline (22 out of 36) developed clinical UMN signs after 1 year (*Χ*^2^ = 4.3, *p* = 0.04), with a positive predictive value of 61%. Finally, when combining the clinical findings both at baseline and at follow-up, 90% (124 out of 138) of patients with MEPs alteration at baseline showed clinical UMN signs at limbs at 1 year, increasing the positive predictive value to 90% (*X*^2^ = 6.6, *p* = 0.01), with a sensitivity of 82% and a specificity of 42%.

**Table 3:** Distribution of clinical and electrophysiological evidences of UMN involvement at presentation

	Patients with clinical evidence of UMN involvement at limbs (*n* 126)	patients without clinical signs of UMN involvement at limbs (*n* 50)
Patients with abnormal MEPs (*n* 138)	102	36
Patients with normal MEPs (*n* 38)	24	14

*X*^2^ = 0.136, *p* = n.s

Furthermore, we evaluated TMS findings among 40 cases clinically classified at baseline as suspected ALS according to EEC, actually LMN phenotype. We found that 68% (*n* = 27) of these patients had TMS abnormalities, while 32% (*n* = 13) remained purely LMN phenotype. About one half of suspected ALS showed MEPs abnormalities in one region (*n* = 19) and another 20% in two regions (*n* = 8).

We also performed a comparative analysis of the distribution of delayed CMCT and waveform abnormalities between the LMN phenotype and the group of patients with UMN signs in at least one region (*n* = 136). Waveform abnormalities were less frequent in the group of patients with LMN phenotype [40% (*n* = 16) versus 62% (*n* = 84), *X*^2^ = 6, *p* = 0.01], whereas no difference in delayed CMCT was observed in the two groups of patients [38% (*n* = 15) versus 36% (*n* = 48)]. LMN phenotype had also significantly higher delayed CMCT in lower limbs [40% (6 out of 15)] compared to the group with clinical UMN signs [10% (5 out of 48), *X*^2^ = 8.3, *p* = 0.02, Table 4]. On the other hand, the distribution of waveform abnormalities among limbs did not differ between the two groups (data not shown).

**Table 4:** Distribution of delayed CMCT in upper, lower and four limbs in patients with abnormal MEPs stratified for phenotype

	Patients with delayed CMCT in upper limbs (37)	Patients with delayed CMCT in lower limbs (11)	Patients with delayed CMCT in four limbs (15)
UMN phenotype (48)	29	5	14
LMN phenotype (ALS suspected) (15)	8	6	1

*X*^2^ = 8.3, *p* = 0.02

After 1 year of follow-up, according to EEC, 46% of patients (*n* = 81/176) were categorized as definite ALS, 27% as probable (*n* = 48/176), 15% as possible (*n* = 26/176) and 12% as suspected (*n* = 21/176) (Table 2). Therefore, ~ 70% of LMN phenotype with MEPs abnormalities at baseline (19 out of 27) developed UMN signs and became clinically ALS within 1 year.

We also evaluated the occurrence of MEP abnormalities stratifying patients according to the site of symptoms onset; we found that the proportion of patients presenting TMS abnormalities in at least one region in the absence of clinical UMN signs at limbs was similar between spinal [72% (*n* = 28/39)] and bulbar-onset ALS [71% (*n* = 8/11)]. Among spinal onset-ALS (*n*.133), more than the 80% of the cases that developed at 1-year clinical evidence of UMN sign (*n* = 51/62) presented TMS abnormalities at baseline in the same region, compared to 50% of bulbar ALS (11 out of 23).

## Discussion

In the present study, we found that TMS shows an overall high sensitivity (82%) and positive predictive value (90%) in identifying subclinical UMN involvement in ALS. At the time of the diagnosis, ~ 70% of our patients presented abnormal MEPs in absence of clinical UMN signs at limbs. This is a relevant finding since the clinical identification of UMN signs may be difficult at the beginning of the disease or in the presence of contemporary LMN involvement in the same districts [7, 13].

Based on the results of our study, the inclusion of MEPs in the ALS diagnostic assessment could help to detect preclinical UMN signs and, therefore, to avoid delay in ALS diagnosis, particularly among LMN phenotype. Indeed, considering MEPs alterations as indicator of preclinical UMN damage, the ALS diagnostic category level could potentially upgrade and interestingly about the 70% of LMN phenotype could receive a diagnosis of ALS at baseline, while they remained not classifiable as ALS according to both AHC and AC. In agreement with this observation, the large part of cases with LMN phenotype and abnormal MEP at baseline developed clinical UMN signs within 1 year.

On the other hand, our data reported a low specificity of TMS (42%) which has also been indicated by clinical neurophysiologists in the Awaji criteria because of the specific way EMG data contributed to the diagnosis [14]. Nevertheless, this study is not intended to demonstrate a high specificity, considering that to date the diagnosis of ALS still results from the combination of the observation of clinical progression of signs and symptoms and the exclusion of other causes. Moreover, the number of potential false positive in our case series is presumed to be very low as an alternative diagnosis was ruled out in all cases after a careful diagnostic work up. The usefulness of MEPs in ALS diagnosis among LMN phenotype is particularly relevant in our case sample. Indeed, the prevalence of suspected ALS is ~ 20%, higher than in other ALS populations and consistent with previous findings in our historical ALS population-based cohort [15]. In that study, we observed that the suspected ALS had a median survival time and a 4-year survival rate not different from that of definite ALS [15]. Moreover, there are several epidemiological data indicating that the disease progression may not proceed in a manner that leads to the evolution of patients through all the diagnostic categories and approximately one quarter of patients with ALS may progress to death from ALS even though their diagnostic category did not change [14, 16]. Therefore, a recent consensus conference sponsored by the International Federation of Clinical Neurophysiology proposed new indications for ALS diagnosis criteria. Among the minimum necessary abnormalities to perform a diagnosis of ALS, the consensus included the observation of lower motor neuron signs in at least two regions again [14].

In disagreement with a previous study [17], TMS detected preclinical UMN involvement more frequently in spinal-onset (80%) than in bulbar-onset (50%) patients, and this could be related to the lack of examination of the bulbar region by our routinely TMS. Another possible explanation of this finding may be the low prevalence (< 30%) at presentation of bulbar UMN signs, which was consistent with a previous population-based observation in our region [18]. However, although the clinical evaluation has been performed by an experienced in ALS, the low frequency of bulbar UMN signs at presentation could be related to a clinical underestimation, and this suggests that routinely MEPs should be performed also in bulbar region to empower the ALS diagnostic certainty.

All these results support that MEP alterations could be used as a reliable marker of preclinical UMN damage and could be incorporated into the Awaji criteria or in the new sets of criteria to allow an early diagnosis and the inclusion in clinical trials [14]. In the present work, we did not perform a survival analysis, according to distribution of MEPs abnormalities, even if a possible evolution of the study will address this topic.

Our study has some limitations, including the already mentioned lack of bulbar region examination with TMS, the ALS tertiary center rather than a population-based cohort design; the lack of EMG and TMS controls after 1 year of follow-up. However, in the absence of serial EMG and TMS, we revaluated the patients using EEC at 1 year, considering these criteria reliable, because of the low risk of false positive [19]. Another limitation is the use of a focal single coil that might reduce the field penetration compared to round or double cone TMS coils, which in turn may induce higher and wider spread electrical fields in superficial cortical regions, and could have been preferable particularly for eliciting lower limbs [20]. Consistently with this observation, about one third of patients with UMN signs had normal MEPs: this finding could be explained either by the low sensibility of the TMS with our methodology or by the lack of specificity of some of the UMN signs, as hyperreflexia or normal deep tendon reflexes in wasted limb.

We, however, believe that these limitations did not significantly biased our results, indicating that TMS has a good positive predictive value to detect UMN damage early in the course of the disease. It can be stated that the presence of altered MEPs in a widespread LMN disease (i.e., two or more body regions) could be sufficient for the diagnosis of ALS even in absence of clinical evidence of UMN signs. This opens to the opportunity that restricted phenotypes of motor neuron diseases can be included in the diagnosis of ALS and, where appropriate, enrolled in RCT.
